# Tricyclic antidepressants induce liver inflammation by targeting NLRP3 inflammasome activation

**DOI:** 10.1186/s12964-023-01128-x

**Published:** 2023-05-25

**Authors:** Wenqing Mu, Guang Xu, Zhilei Wang, Qiang Li, Siqiao Sun, Qin Qin, Zhiyong Li, Wei Shi, Wenzhang Dai, Xiaoyan Zhan, Jiabo Wang, Zhaofang Bai, Xiaohe Xiao

**Affiliations:** 1 Department of Hepatology, the Fifth Medical Center of PLA General Hospital, Beijing, 100039 China; 2 State Key Laboratory of Radiation Medicine and Protection, Institutes for Translational Medicine, Soochow University, Suzhou, 215123 Jiangsu China; 3 School of Traditional Chinese Medicine, Capital Medical University, Beijing, 100069 China; 4 Military Institute of Chinese Materia, Fifth Medical Center of Chinese PLA General Hospital, Beijing, 100039 China; 5 TCM Regulating Metabolic Diseases Key Laboratory of Sichuan Province, Hospital of Chengdu University of Traditional Chinese Medicine, Chengdu, 610072 China

**Keywords:** NLRP3 inflammasome, Idiosyncratic hepatotoxicity, Antidepressant, Tricyclic antidepressant, Nortriptyline

## Abstract

**Background:**

Idiosyncratic drug-induced liver injury (IDILI) is common in hepatology practices and, in some cases, lethal. Increasing evidence show that tricyclic antidepressants (TCAs) can induce IDILI in clinical applications but the underlying mechanisms are still poorly understood.

**Methods:**

We assessed the specificity of several TCAs for NLRP3 inflammasome via MCC950 (a selective NLRP3 inhibitor) pretreatment and *Nlrp3* knockout (*Nlrp3*^*−/−*^) BMDMs. Meanwhile, the role of NLRP3 inflammasome in the TCA nortriptyline-induced hepatotoxicity was demonstrated in *Nlrp3*^*−/−*^ mice.

**Results:**

We reported here that nortriptyline, a common TCA, induced idiosyncratic hepatotoxicity in a NLRP3 inflammasome-dependent manner in mildly inflammatory states. In parallel in vitro studies, nortriptyline triggered the inflammasome activation, which was completely blocked by *Nlrp3* deficiency or MCC950 pretreatment. Furthermore, nortriptyline treatment led to mitochondrial damage and subsequent mitochondrial reactive oxygen species (mtROS) production resulting in aberrant activation of the NLRP3 inflammasome; a selective mitochondrial ROS inhibitor pretreatment dramatically abrogated nortriptyline-triggered the NLRP3 inflammasome activation. Notably, exposure to other TCAs also induced aberrant activation of the NLRP3 inflammasome by triggering upstream signaling events.

**Conclusion:**

Collectively, our findings revealed that the NLRP3 inflammasome may act as a crucial target for TCA agents and suggested that the core structures of TCAs may contribute to the aberrant activation of NLRP3 inflammasome induced by them, an important factor involved in the pathogenesis of TCA-induced liver injury.

Video Abstract

**Supplementary Information:**

The online version contains supplementary material available at 10.1186/s12964-023-01128-x.

## Background

The extensive application of antidepressants in medical field has generated concerns about their toxicity and safety. As reported, almost all antidepressant agents can lead to liver injury, and they are usually idiosyncratic and unpredictable [1, 2]. The hepatotoxicity of antidepressants includes various biological and clinical manifestations ranging from an increase in hepatic enzyme levels to more specific symptoms such as jaundice, loss of hepatocellular functions, and acute liver failure [2]. Tricyclic antidepressants (TCAs), one of the earliest antidepressants classes, have been reported to induce liver toxicity [3, 4]. Similarly, jaundice, aminotransferase abnormalities, and acute hepatitis are common biological and clinical presentations of hepatotoxicity induced by TCAs [5–7]. Simultaneously, evidence suggest that the production of toxic metabolites and eosinophilic infiltration of the liver may cause TCA-induced hepatotoxicity [8–10]. Nortriptyline, a common TCA, has been identified to be hepatotoxic in vitro, causing damage to rat hepatocytes and inducing the leakage of cytoplasmic and lysosomal enzymes [11]. In vivo, the alterations in aspartate transaminase (AST) and alanine aminotransferase (ALT) levels have been reported after long-term administration of nortriptyline [12, 13]. However, the exact molecular mechanisms of liver injury induced by nortriptyline or TCAs still in general need to be determined.

NLRP3 inflammasome, a platform of a macromolecular protein complex, can sense various danger signals responsible for caspase-1 maturation and the subsequent secretion of cytokine interleukin (IL)-1β and gasdermin D (GSDMD)-mediated pyroptosis [14–17]. Although the NLRP3 inflammasome plays an important role in host immune defenses [18–20], evidence show that its aberrant activation is also associated with the pathogenesis of multiple inflammatory and autoimmune diseases including atherosclerosis, type 2 diabetes, gout, as well as cryopyrin-associated periodic syndromes (CAPS) [21–24]. Increasing evidence also suggest that the aberrant activation of the NLRP3 inflammasome is related to hepatotoxicity [25]. For example, nanoparticles are widely used in biological fields and have been demonstrated to induce hepatic injury by initiating the NLRP3 inflammasome activation [26]. Additionally, there are also several therapeutic drugs such as antiepileptic agent carbamazepine [27] and antituberculosis drugs isoniazid and rifampicin [28], induce idiosyncratic drug-induced liver injury (IDILI) by enhancing the NLRP3 inflammasome activation.

In this study, we demonstrated that the TCA agent nortriptyline induced liver toxicity by activating the NLRP3 inflammasome. Additionally, exposure to multiple TCAs that have been reported to induce hepatic injury, including imipramine [11], amitriptyline [5] and protriptyline [29], also triggered its aberrant activation and subsequent downstream effector cytokine generation. These data indicate that the NLRP3 inflammasome plays an important role in TCA-induced idiosyncratic hepatotoxicity and that the NLRP3 inflammasome will be a potential target for the treatment of patients with liver injury caused by TCA agents.

## Materials and methods

### Animal

Wild type (WT) C57BL/6 mice eight weeks of age (female) were obtained from SPF Biotechnology Co., Ltd (Beijing, China). *Nlrp3* knockout (*Nlrp3*^*−/−*^) mice were a generous gift from Dr. Tao Li (National Center of Biomedical Analysis, NCBA, Beijing, China). Animals were raised under pathogen-free and alternating dark/light conditions at 20**–**24 °C.

### Antibodies and reagents

Dulbecco’s modified Eagle’s medium (DMEM) was obtained from MACGENE (Beijing China). Opti-MEM medium and fetal bovine serum (FBS; 04–001-1ACS) were from Gibco (USA). Mouse macrophage colony-stimulating factor (MCS-F; HY-P7085), JC-1 Mitochondrial Membrane Potential Assay Kit (HY-K0601), MCC950 (HY-12815A), N-acetylcysteine (NAC; HY-B0215), Mito-TEMPO ( HY-112879), nortriptyline (HY-B1417), protriptyline (HY-B0949), amitriptyline (HY-B0527A), mirtazapine (HY-B0352), agomelatine (HY-17038), imipramine (HY-B1490), doxepin (HY-B0725), clomipramine (HY-B0457), and phenothiazine (HY-Y0055) were obtained from MedChemExpress (MCE, NJ, USA). Synthetic oligodeoxynucleotide (ODN; tlrl-ttag151) was from InvivoGen (San Diego, USA). MitoSOX™ Red mitochondrial superoxide indicator was provided by Invitrogen (Carlsbad, CA, USA). Anti-mouse caspase-1 (AG-20B-0042-C100) and anti-NLRP3 (AG-20B-0014-C100) were obtained from Adipogen (San Diego, USA). Anti-mouse IL-1β (AF-401-NA) and mouse-IL-1β (SMLB00C) ELISA assay kit were from R&D (Minnesota, USA). Anti-mouse GSDMD (ab209845) and disuccinimidyl suberate (DSS; ab141274) were from Abcam (Cambridge, UK). Anti-ASC (A1170) was purchased from ABclonal (Wuhan, China). Anti-GAPDH (60004–1-1 g) was purchased from Proteintech (Chicago, USA). Mouse-TNF-α (1217202) ELISA assay kit and mouse IL-6 (1210602) ELISA assay kit were obtained from DAKEWE (Beijing, China). GOT (C010-2–1) and GPT (C009-2–1) kits were obtained from Nanjing Jiancheng Bioengineering Institute (Nanjing, China).

### Cell culture and inflammasome activation assay

Bone marrow cells were collected from WT mice or *Nlrp3*^*−/−*^ mice, and then incubated in DMEM containing 1% penicillin/streptomycin and 10% FBS. Furthermore, murine recombinant MCS-F (50 ng/mL) were added to differentiate cells into bone marrow-derived macrophages (BMDMs). Similarly, hepatocytes and Kupffer cells were isolated from WT mice and cultured in low-glucose DMEM (1.0 g/L glucose) and RPMI-1640 medium (10% FBS, 1% penicillin/streptomycin), respectively.

To trigger inflammasome activation, WT or *Nlrp3*^*−/−*^ BMDMs were incubated at a density of 1.2 × 10^6^ cells/mL in 12-well plates. Next, cells were treated with 50 ng/mL ultrapure LPS ( InvivoGen, USA). Subsequently, DMEM was switched to Opti-MEM (in order to avoid FBS in DMEM affecting the results of western blotting and the possible interaction between drugs and proteins) containing nortriptyline (40 μM), protriptyline (40 μM), amitriptyline (40 μM), imipramine (40 μM), mirtazapine (40 μM), agomelatine (40 μM), doxepin (40 μM), clomipramine (40 μM), and phenothiazine (40 μM), and stimulated for twelve hours followed by detection of inflammasome activation. Additionally, BMDMs were primed with LPS followed by MCC950 (2 μM) or ODN (40 μM) treatment for one hour, and eventually stimulated with antidepressants for twelve hours. Likewise, BMDMs, hepatocytes or Kupffer cells were incubated at 6-well plates, and then treated with LPS, MCC950, and nortriptyline. The proteins of the cell supernatants were precipitated and then evaluated by western blot.

### Caspase activity

The experimental protocol for the detection of caspase-1 activity was similar as described previously [30, 31]. Briefly, a Caspase-Glo-s1® Inflammasome Assay kit (Promega, Beijing, China) was applicated to evaluate the caspase-1 activity of cell supernatants and mouse serum, according to the manufacturer's instructions.

### LDH release

The LDH cytotoxicity assay kits (Promega, Madison, USA) were used to assess the release of LDH in cell supernatants.

### Immunoblotting assay

The protein extraction in cell supernatants and immunoblot analysis were performed as described previously [32].

### Enzyme-linked immunosorbent assay (ELISA)

The production of IL-1β, IL-6, and TNF-α in cell supernatants or mouse serum were detected using the corresponding ELISA kits.

### ASC oligomerization assay

BMDMs in a 12-well plate were treated with LPS followed by nortriptyline (10, 20, 40 μM) stimulation for twelve hours. Next, the cell culture supernatants were discarded and 200 μL Triton buffer [33] (1% EDTA-free protease inhibitor cocktail) was added to Lyse these cells. After centrifuging for fifteen minutes (4 °C, 6500 g), the supernatants were collected as the whole cell lysate, the pelleted cells were washed and then resuspended in 200 μL PBS and cross-linked with 4 mM DSS at 37 °C for thirty minutes. After centrifugation under the above conditions, the cross-linked pellets were collected and dissolved for immunoblot analysis.

### Mitochondrial reactive oxygen species (mtROS) measurement

BMDMs were seeded in culture dish tubes (100 mm diameter) overnight, and then pretreated with LPS. Next, the cells were transferred to tubes (1.5 mL) and stimulated with nortriptyline (10, 20, 40 μM). Subsequently, the samples were washed using Hank’s balanced salt solution (HBSS) for twice and then incubated with 4 mM MitoSOX Red mitochondrial superoxide dismutase indicator for ten minutes. Finally, the cells were washed and resuspended in 200 μL of HBSS and then mtROS production was assessed using flow cytometry.

### Measurement of mitochondrial damage

The damage of mitochondrial was evaluated by JC-1 mitochondrial membrane potential assay kits. Briefly, BMDMs were incubated in diameter culture dish tubes (100 mm) and then primed with LPS followed by nortriptyline treatment for six hours (in 1.5 mL tubes). Next, the BMDMs were stained with JC-1 (10 μM) for twenty minutes at 37 °C. After washing and resuspension with PBS, flow cytometry was used to evaluate mitochondrial damage.

### Determination of intracellular potassium

The method for intracellular K^+^ detection has been described previously [34]. Briefly, BMDMs in a 24-well plate were pretreated with LPS and then the DMEM was switched to Opti-MEM medium containing nortriptyline (40 μM) and stimulated for 6 h. After discarding the cell culture medium, BMDMs were washed four times with saline, and then ultrapure HNO_3_ was added to lyse them. Subsequently, collected the samples and boiled for 30 min at 105 °C, and inductively coupled plasma mass spectrometry (ICP-MS) was applicated to detect intracellular K^+^.

### Ca^2+^ analysis

The protocol for Ca^2+^ analysis was described previously [33, 35]. Briefly, BMDMs (5 × 10^5^ cells/mL) were incubated in a 384-well plate overnight followed by LPS pretreatment and then stimulated with nortriptyline. A trace showing nortriptyline-triggered Ca^2+^ mobilization was detected via the FLIPRT Tetra system (Molecular Devices, CA, USA).

### AST and ALT measurement

The levels of AST and ALT in mouse serum were determined using the GOT and GPT assay kits.

### In vivo experiments

Eight-week-old WT or *Nlrp3*^*−/−*^ C57BL/6 mice (female) were first treated with LPS (2 mg/kg) or PBS vehicle via tail vein. After two hours, nortriptyline (20 mg/kg or 40 mg/kg) was administered via intraperitoneal injection and treated for six hours. Then, the mouse serum and liver tissues were collected.

In the second experiment, WT C57BL/6 mice eight weeks of age (female) were treated with 2 mg/kg LPS or PBS vehicle via the tail vein. Two hours later, mice were injected intraperitoneally with nortriptyline (10 mg/kg or 20 mg/kg) or PBS. Six hours after nortriptyline administration, the liver tissues and mouse serum were collected.

In the third experiment, WT C57BL/6 mice (eight-week-old, female) were injected intraperitoneally with MCC950 (50 mg/kg) or PBS before mice were injected via the tail vein with 2 mg/kg LPS. Two hours later, mice were stimulated with nortriptyline (20 mg/kg) via intraperitoneal injection. Subsequently, the mouse serum and liver tissues were collected.

### Statistical analyses

GraphPad Prism 6 (GraphPad Software) and Excel were applicated for statistical and analysis. All data were presented as the mean ± SEM. The unpaired Student’s *t*-test (two groups) or One-Way ANOVA followed by the Dunnett's post hoc test (multi groups). Data were regarded as statistically significant when *P < 0.05, **P < 0.01, or ***P < 0.001.

## Results

### Nortriptyline triggers the inflammasome activation accompanied by caspase-1 maturation, IL-1β secretion, and GSDMD cleavage

**Fig. 1:**
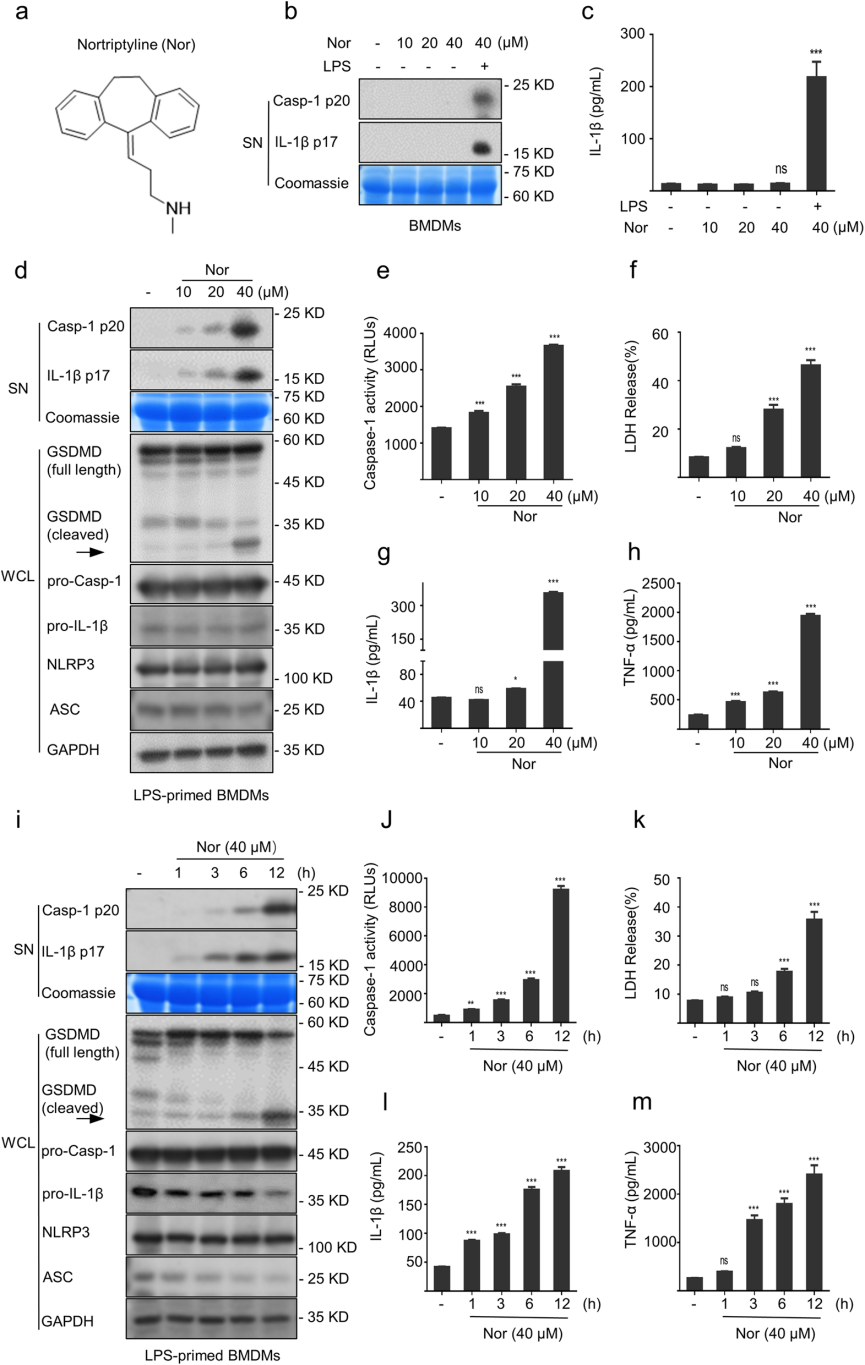
Nortriptyline triggers the inflammasome activation. **a** Nortriptyline structure. **b** and **c** Western blot of IL-1β and caspase-1 (**b**) and ELISA of IL-1β levels (**c**) in culture supernatants (SN) from BMDMs stimulated with nortriptyline in the absence or presence of LPS. **d**–**h** BMDMs were treated with LPS and nortriptyline. Western blot assessed the expression of mature caspase-1 and IL-1β in SN and GSDMD cleavage in whole-cell lysates (WCL) (**d**). The activity of caspase-1 (**e**) and the release of LDH (**f**) were assessed. The levels of IL-1β (**g**) and TNF-α (**h**) were evaluated by ELISA. (**i–m**) Cells were incubated with LPS and then treated with nortriptyline for 1, 3, 6, 12 h, respectively. Western blot evaluated the expression of mature IL-1β and cleaved caspase-1 in SN and GSDMD cleavage in WCL (**i**). The caspase-1 activity (**j**) and LDH (**k**) release in SN. ELISA assay the SN levels of IL-1β (**l**) or TNF-α (**m**). Data are expressed as the mean ± SEM (*n* = 3); *P < 0.05, **P < 0.01, ***P < 0.001 *vs*. the control group; ns, not significant; One-Way ANOVA analysis was followed by the Dunnett's post hoc test

To avoid cytotoxic effect of nortriptyline (Fig. 1a) on inflammasome activation, cell viability was detected. The results suggested that nortriptyline did not show any cytotoxicity in BMDMs at doses below 80 μM after 24 h treatment (Fig. S1a). Next, we evaluated the effect of nortriptyline on inflammasome activation in the presence or absence of LPS. The results indicated that when LPS was used as the priming signal, nortriptyline triggered the activation of caspase-1 and the secretion of IL-1β, while in the absence of LPS, nortriptyline did not trigger the inflammasome activation (Fig. 1b and c). Furthermore, we evaluated the effect of nortriptyline on inflammasome activation in the presence or absence of inflammasome agonists. The results showed that both nortriptyline and carbamazepine enhanced ATP-induced the activation of NLRP3 inflammasome, whereas in the absence of ATP, nortriptyline rather than carbamazepine triggered inflammasome activation (Fig. S2a). Additionally, after BMDMs were treated with LPS and a range of nortriptyline concentrations, as shown in Fig. 1d, e, and g, nortriptyline triggered caspase-1 maturation, IL-1β generation, and GSDMD cleavage, accompanied by lactate dehydrogenase (LDH) release (Fig. 1f), and TNF-α (an inflammasome-independent cytokine) production (Fig. 1h). Similarly, nortriptyline triggered the secretion of these downstream effector cytokines in a time-dependent manner (Fig. 1i–m). These data indicate that nortriptyline triggers the inflammasome activation in a dose- and time-dependent manner.

### Nortriptyline specifically induces NLRP3 inflammasome activation

**Fig. 2:**
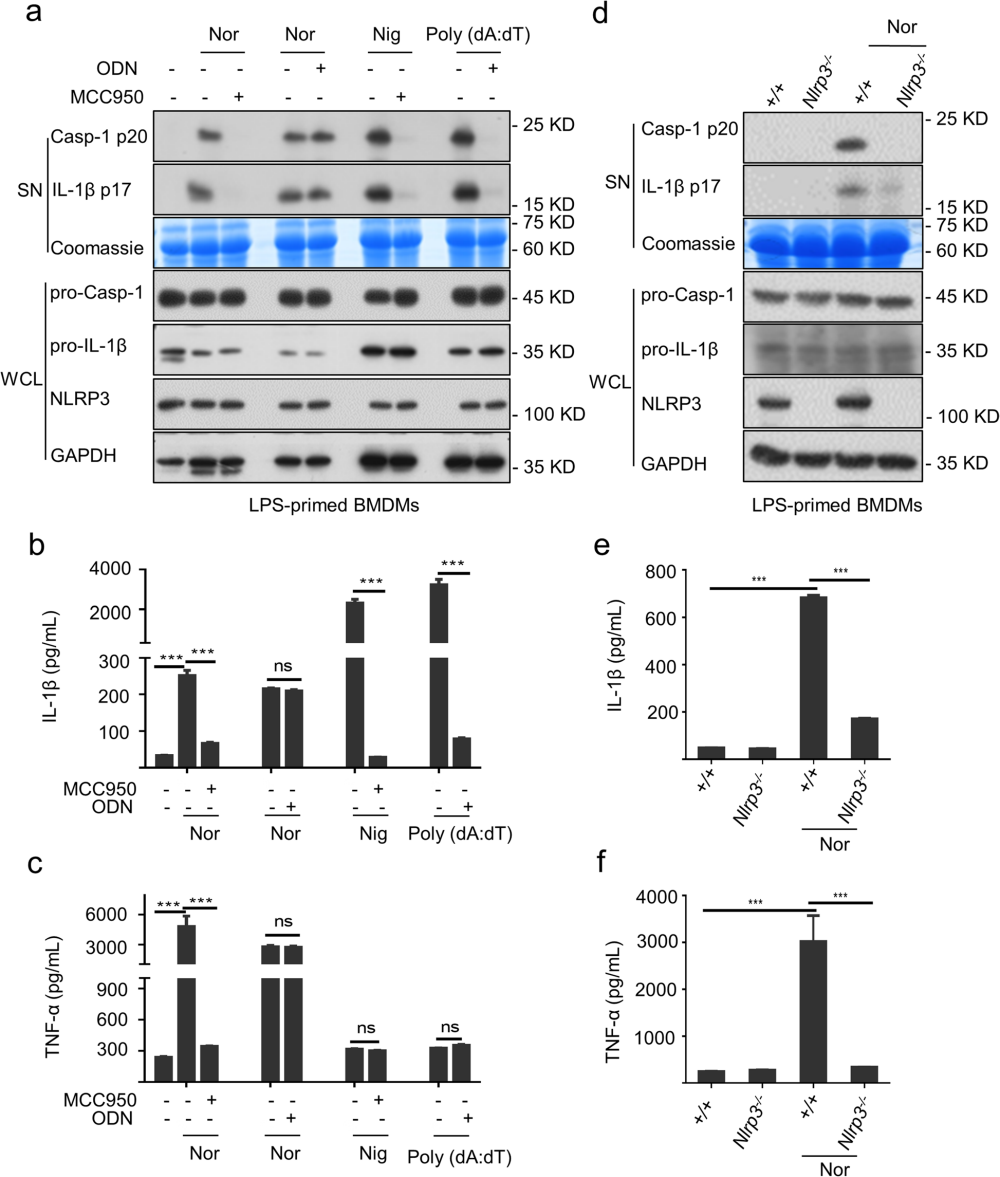
Nortriptyline specifically activates NLRP3 inflammasome, rather than AIM2. **a**-**c** Cells were incubated with LPS and then pretreated with MCC950 or ODN followed by nortriptyline treatment. Western blot assessed the expression of caspase-1 and IL-1β (**a**) in SN. The generation of IL-1β (**b**) or TNF-α (**c**) in SN was detected via the ELISA kit. (**d–f**) *Nlrp3*.^*−/−*^ BMDMs were incubated with LPS and nortriptyline. The expression of IL-1β and caspase-1 in SN (**d**) were evaluated by western blot. The levels of IL-1β (**e**) or TNF-α (**f**) in SN were measured using ELISA kit. Data are presented as the mean ± SEM (*n* = 3); ***P < 0.001; ns, not significant; unpaired Student’s *t*-test (two groups) or One-Way ANOVA followed by the Dunnett's post hoc test (multi groups)

NLRP3, AIM2, and NLRC4 inflammasomes can respond to endogenous or exogenous danger signals, resulting in caspase-1 activation [36, 37]. Our previous studies found that carbamazepine [27] and icariside II [32] promoted NLRP3 inflammasome activation through synergistic effects with ATP or nigericin, thereby inducing IDILI. To identify which type of inflammasome was activated by nortriptyline, LPS-primed BMDMs were treated with MCC950 (a selective NLRP3 inhibitor) [38] and specific AIM2 inhibitor ODN [39] 1 h before nortriptyline stimulation. The results showed that nortriptyline-induced inflammasome activation was completely blocked by the small molecule inhibitor MCC950 as evidenced by the inhibition of caspase-1 maturation as well as IL-1β generation (Fig. 2a and b); the production of TNF-α was also attenuated by MCC950 (Fig. 2c). However, ODN did no effect on nortriptyline-triggered inflammasome activation. These results suggested that nortriptyline induced NLRP3 inflammasome activation rather than AIM2. Furthermore, conditional knockout of *Nlrp3* in LPS-primed BMDMs were applicated to assessed the effects of nortriptyline on inflammasome activation. The data indicated that selective knockout of *Nlrp3* completely abrogated nortriptyline-elicited caspase-1 maturation, IL-1β secretion, and TNF-α generation (Fig. 2d–f). Collectively, these results reveal that nortriptyline specifically targets the NLRP3 inflammasome activation.

### Nortriptyline triggers the NLRP3 inflammasome activation by inducing mitochondrial damage and the subsequent mtROS accumulation

**Fig. 3:**
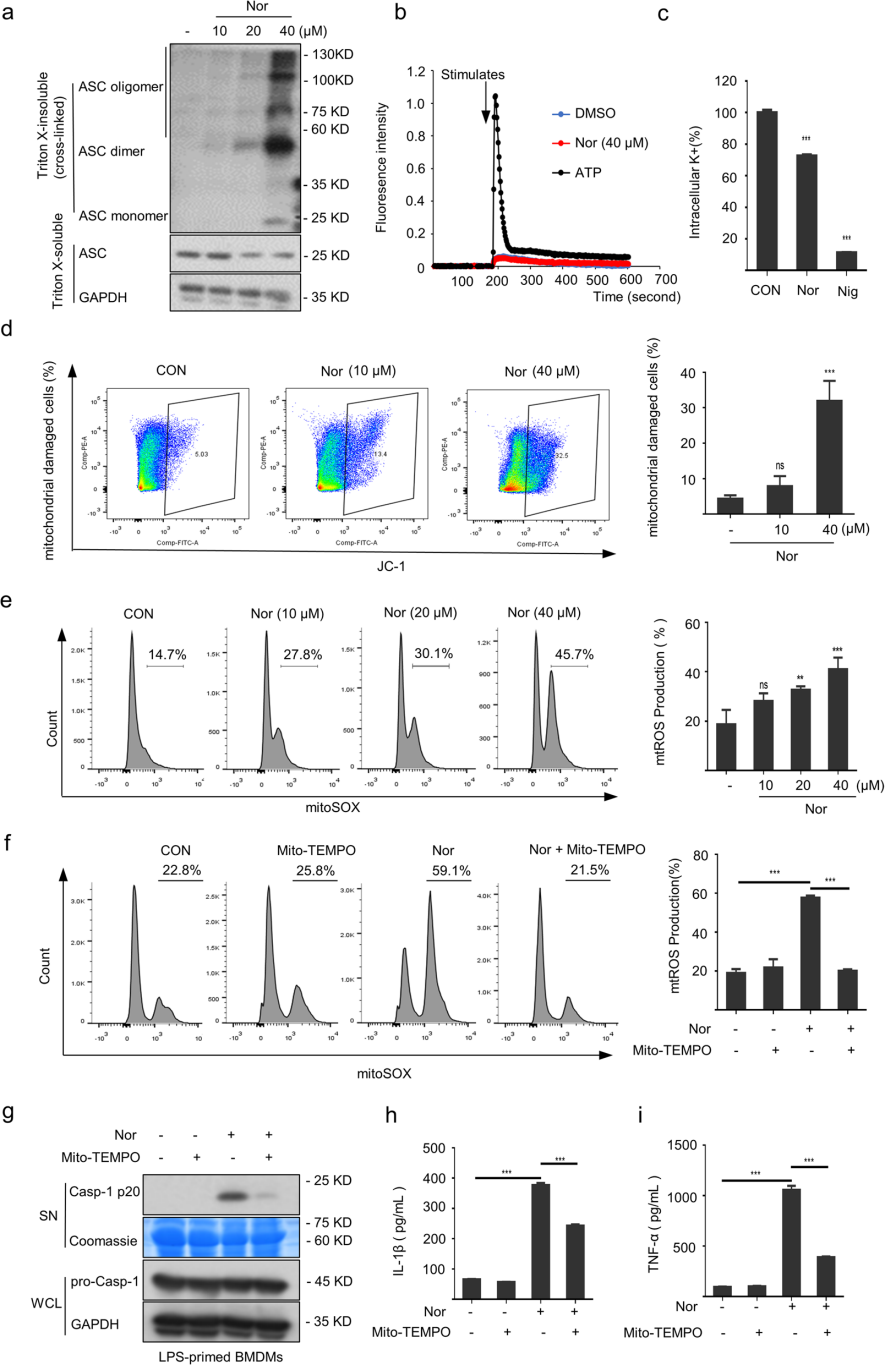
Nortriptyline induces NLRP3-dependent ASC oligomerization by targeting mtROS accumulation but has a weak effect on potassium efflux and no effect on Ca^2+^ mobilization. **a** The ASC oligomerization from BMDMs stimulated with nortriptyline was evaluated by western blot. **b** Mobilization of Ca^2+^. **c** Detection of intracellular K^+^. **d** The mitochondrial damaged cells were determined via JC-1 staining. **e** Cells were treated with LPS and nortriptyline and then the mtROS content was evaluated by flow cytometry. **f**–**i** BMDMs were incubated with LPS and Mito-TEMPO followed by nortriptyline treatment, the content of mtROS (**f**) was evaluated by flow cytometry, the expression of caspase-1 in SN (**g**) was assessed via western blotting and the generation of IL-1β (**h**) or TNF-α (**i**) in SN was detected using ELISA. Data are presented as mean ± SEM (*n* = 3); *P < 0.05, **P < 0.01, ***P < 0.001; ns, not significant; One-Way ANOVA followed by Dunnett's post hoc test

We further investigated the mechanism of how nortriptyline triggered the NLRP3 inflammasome activation. Evidence indicate that ASC oligomerization is a crucial contributor to caspase-1 activation and IL-1β generation [40]. In our work, nortriptyline induced oligomerization of ASC (Fig. 3a), suggesting that nortriptyline acts upstream of ASC oligomerization to trigger the NLRP3 inflammasome activation.

Ca^2+^ mobilization is generally regarded as a crucial upstream event for NLRP3 inflammasome activation [41, 42]. Next, the role of Ca^2+^ influx in nortriptyline-triggered NLRP3 inflammasome activation was assessed. As shown in Fig. 3b, compared with ATP-induced Ca^2+^ mobilization under the same conditions, nortriptyline did not trigger Ca^2+^ influx. Furthermore, to further evaluate the role of Ca^2+^ mobilization in nortriptyline-triggered the NLRP3 inflammasome activation, cells were primed with LPS followed by EDTA (a highly effective Ca^2+^ chelator) pretreatment for 1 h with the aim of chelating extracellular Ca^2+^ and then stimulated with nortriptyline and ATP. The results showed that EDTA (0.625, 1.25, 2.5, 5, 10 mM) dramatically abrogated the NLRP3 inflammasome activation induced by ATP rather than nortriptyline (Fig. S3a). These data indicate that Ca^2+^ influx does not mediate the process of nortriptyline-induced the activation of NLRP3 inflammasome. Additionally, potassium efflux is always considered to act upstream of the NLRP3 activation that precedes NLRP3 inflammasome activation [43]. Our results showed that the potassium efflux triggered by nortriptyline was faint (Fig. 3c).

The mitochondrial damage and accumulation of mtROS is another crucial upstream signal implicated in the NLRP3 inflammasome activation [44, 45]. In our further study, flow cytometry results showed that increased dose of nortriptyline further aggravated the mitochondrial damage (Fig. 3d), indicating that the mitochondrial damage plays an important role in nortriptyline-induced the NLRP3 inflammasome activation. Furthermore, the effect of nortriptyline on the mtROS accumulation was evaluated. The results showed that nortriptyline stimulation induced the mtROS accumulation (Fig. 3e), suggesting that the mtROS production was related to aberrant activation of the nortriptyline-triggered NLRP3 inflammasome. To further investigate the role of mtROS accumulation in nortriptyline-induced NLRP3 inflammasome activation, we pretreated LPS-primed BMDMs with a selective mitochondrial ROS inhibitor Mito-TEMPO [46, 47]. The results showed that Mito-TEMPO (0.5 mM) pretreatment dramatically attenuated mtROS accumulation induced by nortriptyline (Fig. 3f). Meanwhile, the nortriptyline-triggered caspase-1 activation, IL-1β secretion and TNF-α production were also abrogated by Mito-TEMPO pretreatment (Fig. 3g–i). These results suggest that mtROS production plays an crucial role in nortriptyline-initiated NLRP3 inflammasome activation. Additionally, a ROS scavenger NAC [48] was also used to evaluate the effects of mtROS on nortriptyline-triggered NLRP3 inflammasome activation. Consistent with the results of Mito-TEMPO pretreatment, NAC (2.5 mM) treatment completely abrogated nortriptyline-induced mtROS accumulation, secretion of downstream effector cytokines, and TNF-α generation (Fig. S4a–e), which further demonstrated that nortriptyline initiated the NLRP3 activation by triggering mtROS accumulation.

### Multiple TCAs specifically induce the NLRP3 inflammasome activation by triggering upstream signaling events

**Fig. 4:**
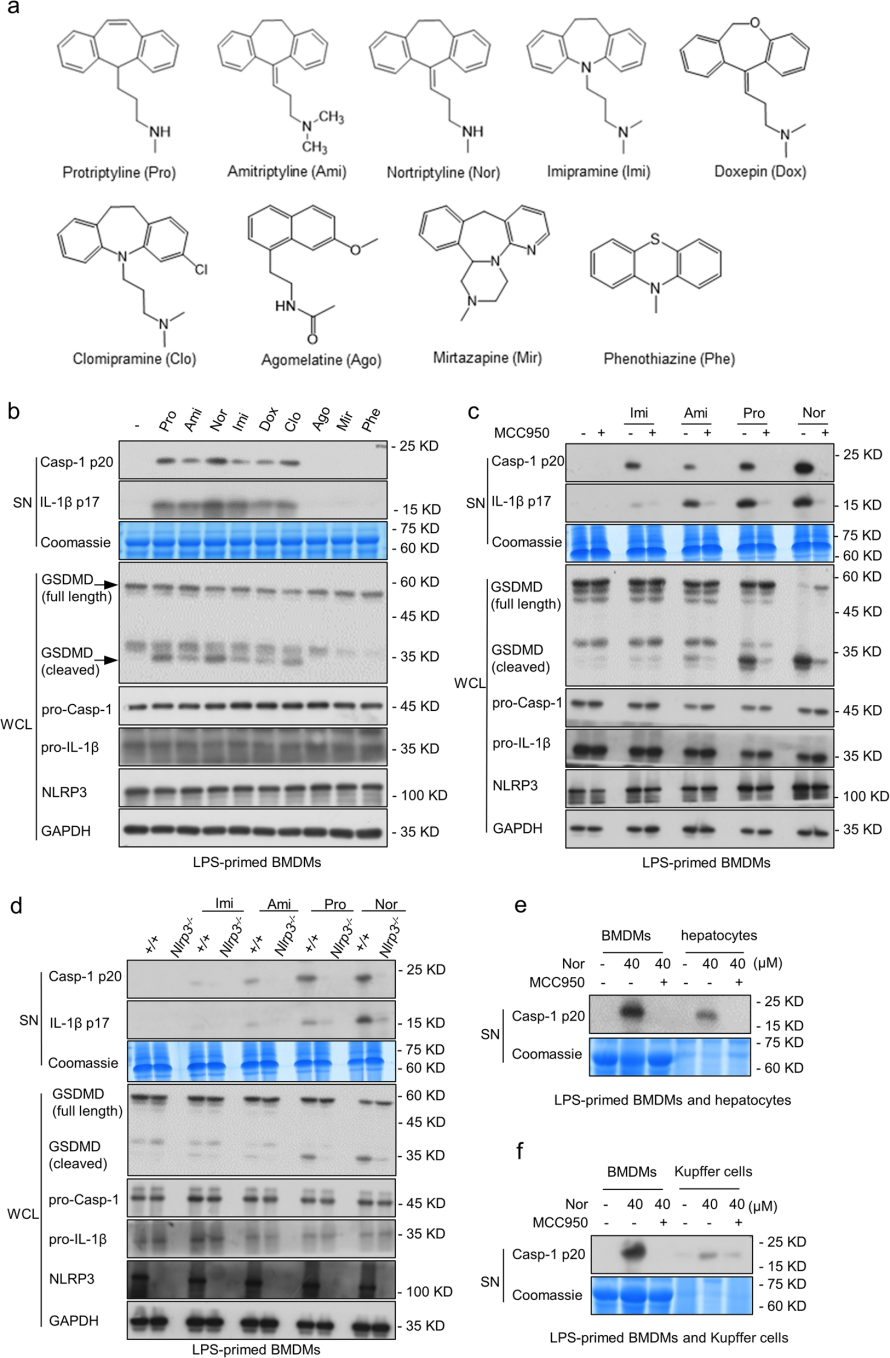
Multiple TCAs specifically induce the NLRP3 inflammasome activation by triggering upstream signaling events. **a** The structures of these antidepressants. **b** LPS-primed BMDMs were stimulated with protriptyline, amitriptyline, nortriptyline, imipramine, doxepin, clomipramine, agomelatine, mirtazapine, and phenothiazine. Western blot assessed the expression of caspase-1 and IL-1β in SN and GSDMD cleavage in WCL. **c** BMDMs were incubated with LPS and MCC950 and then treated with imipramine, amitriptyline, protriptyline and nortriptyline. The expressions of IL-1β and caspase-1 in SN as well as GSDMD cleavage in WCL were evaluated using western blotting. **d** *Nlrp3*^*−/−*^ BMDMs were incubated with LPS followed by imipramine, amitriptyline, protriptyline and nortriptyline stimulation. The expression of IL-1β and caspase-1 in SN and GSDMD cleavage in WCL were measured by western blotting. **e** BMDMs or hepatocytes were pretreated with LPS and MCC950 and then treated with nortriptyline, western blot was used to measure the expression of caspase-1 in SN. **f** BMDMs or Kupffer cells were incubated with LPS and MCC950 followed by nortriptyline treatment. The expression of caspase-1 in SN were assessed by western blot

The TCAs, one of the most common antidepressants classes, and their core structure is usually composed of a seven-membered heterocyclic ring connected with two benzene rings. To investigate the relationship between the structure of TCAs and inflammasome activation, and whether other antidepressants with structures similar to TCAs have an effect on inflammasome activation, we chose six TCAs (protriptyline, amitriptyline, nortriptyline, imipramine, doxepin and clomipramine) and three other antidepressants (agomelatine, mirtazapine, and phenothiazine) with a structure similar to TCAs to explore the effect on inflammasome activation (Fig. 4a). LPS-primed BMDMs were treated with these drugs for 12 h. Western blot analysis showed that the six TCAs could trigger the caspase-1 activation, IL-1β secretion, and GSDMD cleavage, whereas other antidepressants did not induce the inflammasome activation (Fig. 4b).

Next, we selected four common TCAs to further confirm whether these TCAs function by activating the NLRP3 inflammasome. As shown in Fig. 4c and Fig. S5a, the inflammasome activation triggered by imipramine, amitriptyline, protriptyline, and nortriptyline was abrogated by MCC950; a decrease in TNF- α was also observed (Fig. S5b). Notably, knockout of *Nlrp3* in BMDMs resulted in the inhibition of secretion of downstream effector cytokines and GSDMD cleavage, compared with TCAs-treated WT BMDMs (Fig. 4d and Fig. S5c). Similarly, *Nlrp3* deficiency reduced TCA-induced TNF-α production (Fig. S5d). Furthermore, we further evaluated how these TCAs induce the NLRP3 inflammasome activation. The results showed that the activation of NLRP3 inflammasome triggered by these TCAs could be dramatically inhibited by NAC pretreatment, and the high concentration of KCl also had an inhibitory effect on the TCA-induced NLRP3 inflammasome activation (Fig. S6a), suggesting that these TCAs induced the NLRP3 inflammasome activation by triggering upstream signaling events. Collectively, these findings suggest that the NLRP3 inflammasome can be specifically activated when treated with these TCAs. We explored the structural similarities among these compounds and found that the core structure of TCAs may be an important factor in the aberrant activation of NLRP3 inflammasome induced by them.

Additionally, we evaluated the effect of nortriptyline on the NLRP3 inflammasome activation in hepatocytes. Primary hepatocytes and hepatic macrophages (Kupffer cells) were pretreated with LPS and then treated with MCC950 followed by nortriptyline stimulation. Consistent with the results of nortriptyline-triggered NLRP3 inflammasome activation in BMDMs, these data demonstrated that in both hepatocytes and Kupffer cells, nortriptyline triggered the caspase-1 cleavage and IL-1β secretion (Fig. 4e and f. and Fig. S7a and b). Thus, these results suggest that nortriptyline could induce activation of the NLRP3 inflammasome in both macrophages and hepatocytes.

### Nortriptyline induces idiosyncratic hepatotoxicity by triggering NLRP3 inflammasome activation

**Fig. 5:**
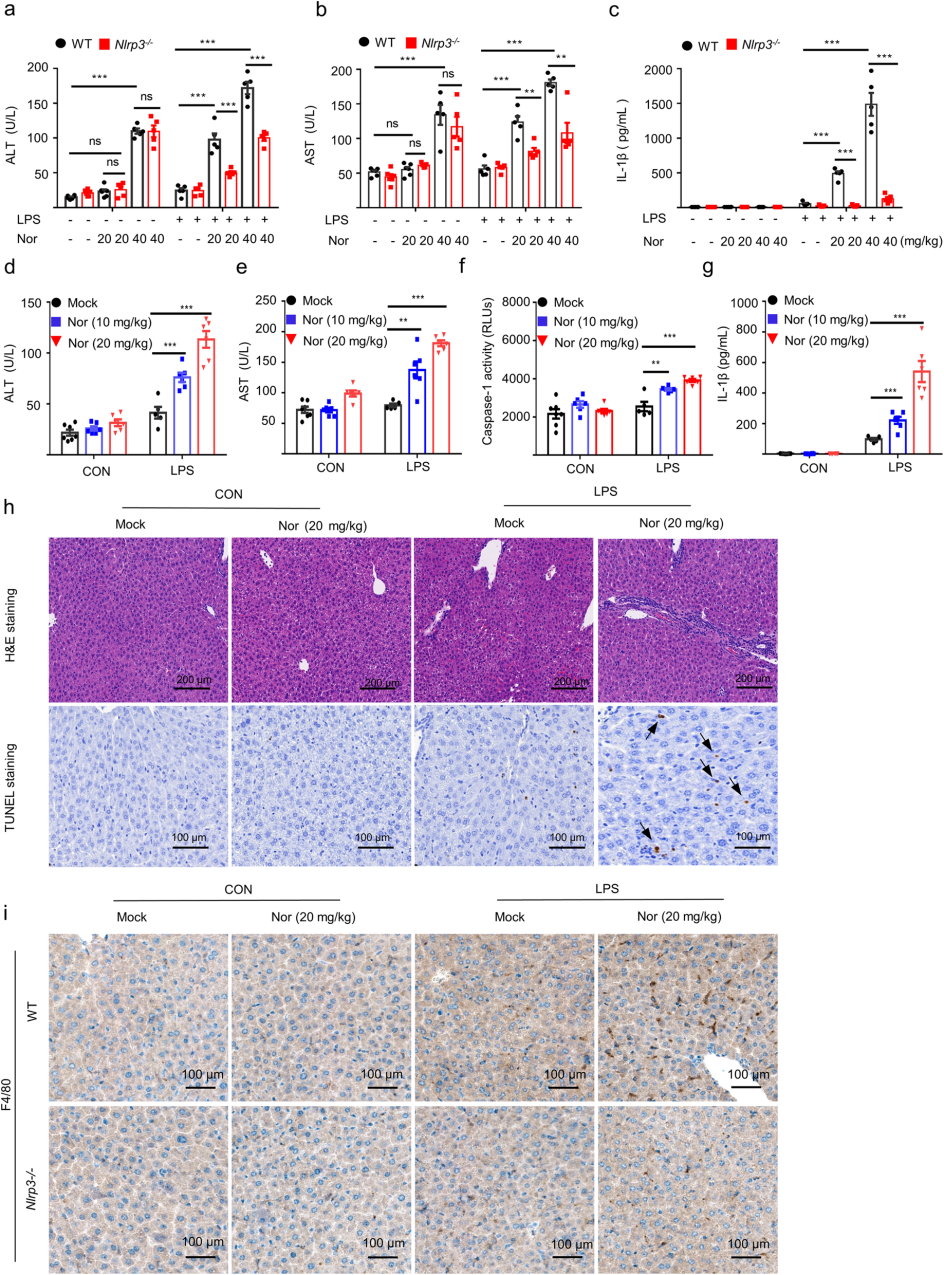
Nortriptyline induces idiosyncratic hepatotoxicity by specifically triggering the NLRP3 inflammasome activation. **a**–**c** WT and *Nlrp3*^*−/−*^ mice were pretreated with LPS and then treated with nortriptyline (WT mice: *n* = 6 control group and nortriptyline (20 mg/kg) group, *n* = 5 other WT groups; *Nlrp3*^*−/−*^ mice: *n* = 6 control group, *n* = 5 other *Nlrp3*^*−/−*^ groups). The levels of serum ALT (**a**), AST (**b**), and IL-1β (**c**) were detected by corresponding assay kits. Data are shown as the mean ± SEM; **P < 0.01, ***P < 0.001; ns, not significant; unpaired Student’s *t-*test. **d–g** WT mice were pretreated with LPS and then treated with different doses of nortriptyline (10 mg/kg, 20 mg/kg; *n* = 7 control group; *n* = 5 LPS group; *n* = 6 other groups). Corresponding assay kits were used to determine the levels of ALT (**d**), AST (**e**), caspase-1 (**f**), and IL-1β (**g**) in mouse sera. Data are shown as the mean ± SEM; *P < 0.05, **P < 0.01, ***P < 0.001 *vs.* LPS group; One-Way ANOVA followed by the Dunnett's post hoc test. **g** TUNEL staining and H&E staining were applied to assess the hepatocyte focal necrosis and inflammatory infiltration. H&E staining: scale bar 200 μm; TUNEL staining: scale bar 100 μm. **h** Representative IHC staining for F4/80 in sections of paraffin‐embedded liver tissues. Scale bar: 100 μm

Next, we evaluated whether the NLRP3 inflammasome activation was involved in nortriptyline-induced hepatotoxicity in vivo. As shown in Fig. 5a–c, in the absence of LPS, low dose of nortriptyline (20 mg/kg) did not induce liver injury, whereas a higher dose of nortriptyline (40 mg/kg) could lead to elevation of the serum ALT and AST in WT mice. Interestingly, this hepatotoxicity could neither induce the secretion of inflammatory cytokine IL-1β nor be blocked by *Nlrp3* deficiency. In the presence of LPS, however, both low dose (20 mg/kg) and high dose (40 mg/kg) of nortriptyline would induce hepatic injury evidenced by the elevation of the serum ALT and AST in WT mice accompanied by up-regulation of the inflammatory cytokine IL-1β, and these changes could be reversed in *Nlrp3*^*−/−*^ mice. These data suggest that the mechanism of nortriptyline-induced hepatotoxicity is different in the presence or absence of LPS. As reported, the TCA agent nortriptyline-driven liver injury is usually idiosyncratic and unpredictable, even at therapeutic doses [1, 2]. Compared with the high dose of nortriptyline-induced liver injury, we think that the low dose of nortriptyline-induced hepatotoxicity under the inflammatory conditions can better simulate the idiosyncratic clinical manifestation. Additionally, there are several studies have shown that the IDILI can be imitated in mice by co-exposure to non-hepatotoxic doses of LPS and drugs that can induce IDILI [49–51]. Therefore, we used LPS to simulate a mild inflammation state at the animal level and further investigated the idiosyncratic hepatotoxicity induced by nortriptyline at a low dose. As shown in Fig. 5d–g, compared with other groups, the combination of LPS and nortriptyline increased the levels of serum AST, ALT, caspase-1 and IL-1β in a dose-dependent manner. Meanwhile, co-exposure to LPS and nortriptyline also triggered the TNF-α production (Fig. S8a), and led to pathological injury, including hepatocyte focal necrosis and inflammatory infiltration (Fig. 5h). In addition, the LPS/nortriptyline-triggered IL-6 secretion and TNF-α production could be significantly improved in the *Nlrp3*^*−/−*^ mice (Fig. S8b and c) and, compared with the control group, nortriptyline group and LPS group, co-treatment with LPS and nortriptyline led to elevation of the F4/80 expression on paraffin-embedded liver sections by IHC, whereas these expressions were dramatically blocked in *Nlrp3*^*−/−*^ mice (Fig. 5i). Taken together, these data suggest that the aberrant activation of the NLRP3 inflammasome may be a crucial contributor to LPS/nortriptyline-induced IDILI.

### MCC950 pretreatment reverses LPS/nortriptyline-induced hepatotoxicity

**Fig. 6:**
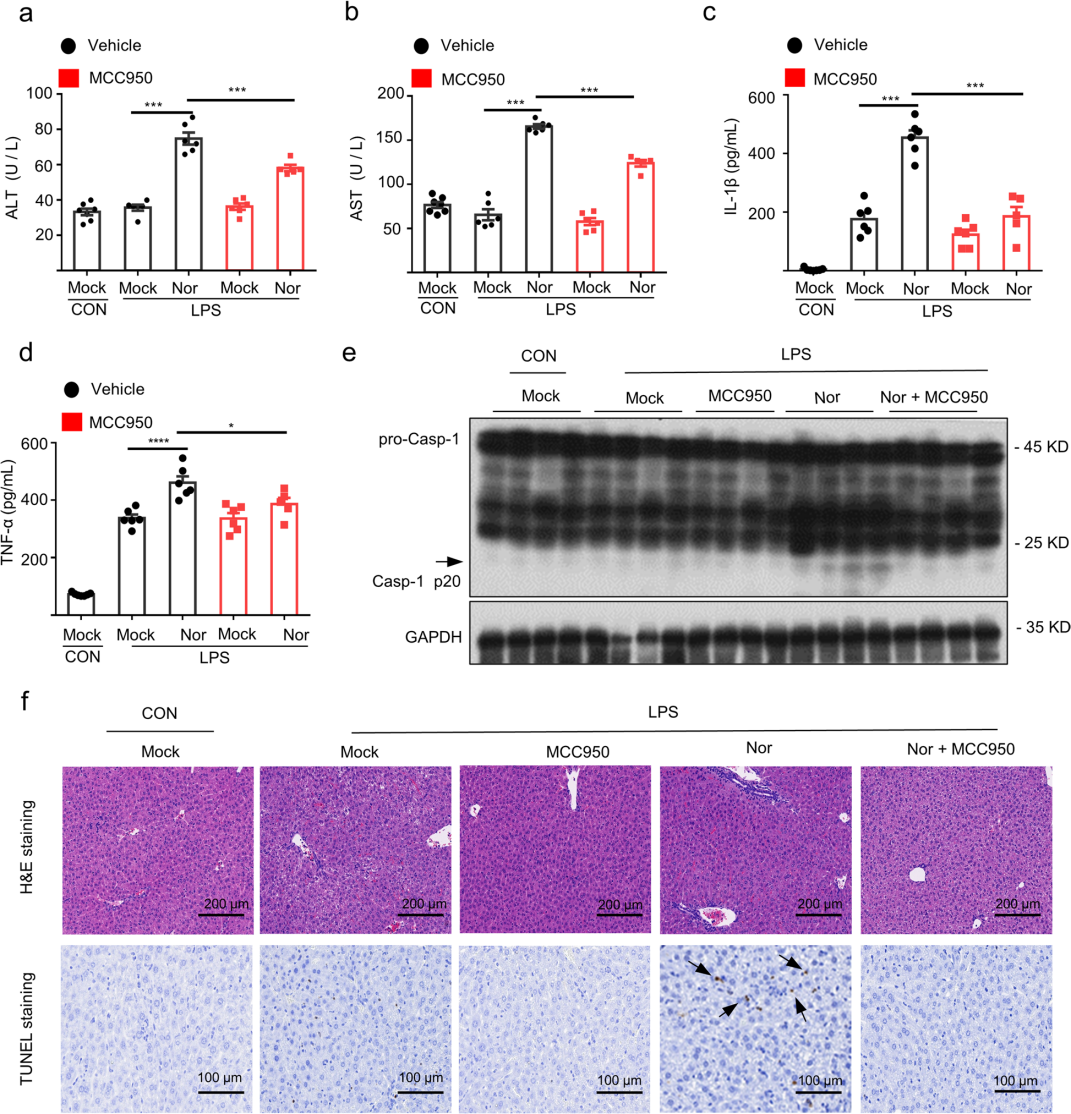
MCC950 pretreatment rescues nortriptyline-driven liver toxicity. **a**–**d** WT mice were pretreated with MCC950 followed by LPS treatment and then stimulated with nortriptyline. Serum levels of ALT (**a**) and AST (**b**) were evaluated by GPT or GOT kits and the serum levels of IL-1β (**c**) and TNF-α (**d**) were detected via ELISA kits. **e** Western blot analysis of caspase-1 activation of the liver tissue. **f** TUNEL and H&E staining were applied to assess inflammatory infiltration and apoptosis in the cells. Data are shown as the mean ± SEM (*n* = 7 control group; *n* = 5 LPS/MCC950/Nor group; *n* = 6 other groups); *P < 0.05, ***P < 0.001 *vs*. LPS/nortriptyline group; One-Way ANOVA followed by Dunnett's post hoc test. H&E staining: scale bar 200 μm; TUNEL staining: scale bar 100 μm

To determine the vital role of the NLRP3 inflammasome in nortriptyline-driven hepatotoxicity, mice were pretreated with MCC950 before administration of LPS and nortriptyline; the levels of serum AST, ALT, IL-1β, as well as TNF-α were measured. As shown, abolishing NLRP3 inflammasome activation by MCC950 reduced LPS/nortriptyline-induced AST and ALT levels. Simultaneously, IL-1β secretion and TNF-α generation were also abrogated by MCC950 (Fig. 6a–d). Moreover, western blot assay suggested that the MCC950 treatment suppressed the expression of caspase-1 (Fig. 6e). Additionally, the small molecular inhibitor MCC950 significantly decreased LPS/nortriptyline-induced inflammatory infiltration and hepatocyte focal necrosis in the liver tissues (Fig. 6f). In summary, these data illustrate that co-treatment with LPS and nortriptyline induces liver injury via NLRP3 inflammasome activation.

## Discussion

IDILI is a severe and unpredictable disease caused by the interaction of host and environmental factors with these drugs. Multiple common clinical antidepressants have been reported to be associated with hepatotoxicity. However, there is a significant gap in our understanding of liver injury induced by antidepressants and underlying molecular mechanisms. In our study, we found that a series of antidepressants including nortriptyline, amitriptyline, imipramine, and protriptyline, initiated the NLRP3 inflammasome activation. In particular, we have demonstrated that nortriptyline triggered the accumulation of mtROS resulting in NLRP3 inflammasome activation, thereby inducing IDILI. These findings suggest that the tricyclic core structure of antidepressants may be a crucial factor involved in the immunoallergic mechanism of the deleterious effect of drugs on the liver induced by triggering the aberrant activation of NLRP3 inflammasome. In support of this hypothesis, antipsychotic phenothiazines and TCA agents amineptine and clomipramine having common tricyclic chemical structure have also been reported to induce liver injury [7, 52–54]. Interestingly, our animal studies showed that the mechanisms of hepatotoxicity induced by a high dose of nortriptyline (40 mg/kg) was different in the presence or absence of inflammatory inducers such as LPS, a lower dose of nortriptyline (20 mg/kg) could induce IDILI in mildly inflammatory states. Collectively, likelihood of IDILI development in TCAs using individuals is higher when they have inflammation related diseases such as bacterial infection or gout.

DAMPs-induced inflammatory responses are independent of pathogen infection and regarded as sterile inflammation, and are key contributor to liver injury [55]. DAMPs can activate innate immune cells, causing to the release of multiple chemokines and cytokines that in turn recruit inflammatory cells and activate immune responses [55, 56]. It has been established that multiple DAMPs such as monosodium urate (MSU) crystals [57], silica [58], alum salt [59] and cholesterol crystals [60] can trigger the assembly and activation of the NLRP3 inflammasome. Similarly, our data showed that TCA agents also induced the assembly and activation of it. This suggests that in certain specific circumstances, TCAs can act as DAMPs, triggering common intracellular molecular signaling or events. After the molecular signaling has been sensed by classical pattern recognition receptors (PRRs) such as NLRP3, TCAs initiate the aberrant activation of NLRP3 inflammasome, thereby inducing inflammation and causing liver injury.

Ion fluxes, the crucial upstream signaling events involved in NLRP3 inflammasome activation [42, 43]. In our study, Ca^2+^ influx was significantly triggered by ATP rather than by nortriptyline, suggesting that TCA-triggered NLRP3 inflammasome activation is independent of Ca^2+^ influx. Additionally, compared with nigericin-induced K^+^ efflux, the changes in potassium ions induced by nortriptyline were scant. Therefore, potassium efflux may play a minor role in TCAs-triggered NLRP3 inflammasome activation. Since ROS generated by mitochondria with reduced membrane potential can initiate NLRP3 inflammasome activation, the accumulation of mtROS into the cytosol is widely recognized as an indispensable upstream event implicated in NLRP3 activation [48]. As one of the earliest established triggers of the NLRP3 inflammasome activation, the production of mtROS can be induced by different agonists. For example, saturated palmitate fatty acid affected the NLRP3 inflammasome activation and triggered inflammatory cytokine secretion in a mtROS-dependent manner [61]. Additionally, conditional deletion of T cell immunoglobulin and mucin-containing molecule 3 (TIM-3) in dendritic cells (DCs) caused increased accumulation of ROS, resulting in NLRP3 inflammasome activation [62]. Similarly, our work showed that nortriptyline triggered the mitochondrial damage and the subsequent production of mtROS, and a selective mitochondrial ROS inhibitor Mito-TEMPO pretreatment dramatically abrogated nortriptyline-induced caspase-1 maturation and IL-1β generation. Our findings suggest that the induction of mtROS production may be a crucial mechanism in the NLRP3 inflammasome activation initiated by TCA agents.

In summary, our study demonstrates that TCAs may act as a DAMP to initiate the aberrant activation of NLRP3 inflammasome under certain circumstances, thereby inducing liver injury. Direct targeting and accumulation of mtROS is a crucial contributor to the TCA-induced NLRP3 inflammasome activation. Additionally, the tricyclic core structure of antidepressants may be conducive to trigger its aberrant activation. Thus, the use of a combination of selective inhibitors of the NLRP3 inflammasome may be a valid therapeutic strategy for treatment of hepatic injury.

## Supplementary Information

**Additional file 1:** Supplementary information on liver injury induced by TCAs by triggering NLRP3 inflammasome activation. **Fig. S1.** Cell viability of BMDMs administrated with nortriptyline was assessed using CellTiter-Glo Assay, which is according to quantitation of ATP. **Fig. S2.** Tricyclic antidepressantnortriptyline triggers the inflammasome activation in the absence of agonists.LPS-primed BMDMs were treated with carbamazepine and nortriptyline for 12 h or treated with them for 1 h followed by ATP stimulation, respectively. Western blotting was used to assess the expression of caspase-1 and IL-1β in SN. **Fig. S3.** Nortriptyline has no effect on Ca^2+^ mobilization.LPS-primed BMDMs were pretreated with EDTAand then stimulated with nortriptyline. Western blotting was used to assess the expression of caspase-1 in SN. **Fig. S4.** Nortriptyline activates NLRP3 inflammasome by inducing the accumulation of mtROS.LPS-primed BMDMs were pretreated with NACfor 1 h and then treated with nortriptyline for 6 h. The content of mtROSwas measured by flow cytometry. Western blotting was used to assess the expression of IL-1β and caspase-1 in cell SN as well as pro-IL-1β, pro-caspase-1, and NLRP3in WCL. The level of caspase-1activity. The levels of IL-1βand TNF-αin SN using ELISA. Data are presented as mean ± SEM; *P < 0.05, ***P < 0.001; One-Way ANOVA followed by Dunnett's post hoc test. **Fig. S5.** Multiple TCAs specifically trigger the aberrant activation of NLRP3 inflammasome.LPS-primed BMDMs were pretreated with MCC950 and then stimulated with Imi, Ami, Pro, and Nor for 12 h, the levels of IL-1βand TNF-αwere detected by ELISA kits.WT and *Nlrp3*^*-/-*^ BMDMs were primed with LPS and then stimulated with Imi, Ami, Pro and Nor, the levels of IL-1βand TNF-αwere evaluated by ELISA kits. Data are expressed as the mean ± SEM,; *P < 0.05, **P < 0.01, ***P < 0.001 *vs.* the control; ns, not significant; unpaired Student’s *t*-testfollowed by the Dunnett's post hoc test. **Fig. S6.** Multiple TCAs induce the NLRP3 inflammasome activation by triggering upstream signaling events.LPS-primed BMDMs were pretreated with MCC950, NAC, and KCl for 1 h followed by these TCAs stimulation, respectively. Western blotting was used to assess the expression of caspase-1in SN. **Fig. S7.** Nortriptyline directly triggered activation of the NLRP3 inflammasome in both hepatocytes and Kupffer cells.LPS-primed BMDMs and hepatocytes were pretreated with MCC950 and then stimulated with nortriptyline for 12 h. The levels of IL-1β in SN were detected using ELISA kits.BMDMs and Kupffer cells were primed with LPS and then pretreated with MCC950 followed by nortriptyline stimulation for 12 h. The levels of IL-1β in SN were detected using ELISA kits. Data are presented as the mean ± SEM; ***P < 0.001; One-Way ANOVA followed by the Dunnett's post hoc test. **Fig. S8.** Nortriptyline induces IDILI by triggering the activation of NLRP3 inflammasome.Female WT C57BL/6 mice were pretreated with LPSand then treated with different doses of nortriptyline. The serum levels of TNF-α were detected by ELISA kits.WT or *Nlrp3*^*-/-*^ mice were pretreated with LPS and then treated with nortriptyline. ELISA kits were used to determine the levels of IL-6and TNF-αin mouse sera. Data are shown as the mean ± SEM; ***P < 0.001; ns, not significant. Statistics differences were analyzed using an unpaired Student’s t-test.

**Additional file 2:** Raw data.

## Data Availability

The datasets used and analyzed during the current study are available from the corresponding author on reasonable request.
