# Supplementary material for: Tricyclic antidepressants induce liver inflammation by targeting NLRP3 inflammasome activation
Source: Cell Commun Signal. 2023 May 25;21:123. doi: 10.1186/s12964-023-01128-x (PMC10214596; doi:10.1186/s12964-023-01128-x)
Supplement: Supplementary file 2 — Additional file 1: Supplementary information on liver injury induced by TCAs by triggering NLRP3 inflammasome activation. Fig. S1. Cell viability of BMDMs administrated with nortriptyline was assessed using CellTiter-Glo Assay, which is according to quantitation of ATP. Fig. S2. Tricyclic antidepressantnortriptyline triggers the inflammasome activation in the absence of agonists.LPS-primed BMDMs were treated with carbamazepine and nortriptyline for 12 h or treated with them for 1 h followed by ATP stimulation, respectively. Western blotting was used to assess the expression of caspase-1 and IL-1β in SN. Fig. S3. Nortriptyline has no effect on Ca2+ mobilization.LPS-primed BMDMs were pretreated with EDTAand then stimulated with nortriptyline. Western blotting was used to assess the expression of caspase-1 in SN. Fig. S4. Nortriptyline activates NLRP3 inflammasome by inducing the accumulation of mtROS.LPS-primed BMDMs were pretreated with NACfor 1 h and then treated with nortriptyline for 6 h. The content of mtROSwas measured by flow cytometry. Western blotting was used to assess the expression of IL-1β and caspase-1 in cell SN as well as pro-IL-1β, pro-caspase-1, and NLRP3in WCL. The level of caspase-1activity. The levels of IL-1βand TNF-αin SN using ELISA. Data are presented as mean ± SEM; *P < 0.05, ***P < 0.001; One-Way ANOVA followed by Dunnett's post hoc test. Fig. S5. Multiple TCAs specifically trigger the aberrant activation of NLRP3 inflammasome.LPS-primed BMDMs were pretreated with MCC950 and then stimulated with Imi, Ami, Pro, and Nor for 12 h, the levels of IL-1βand TNF-αwere detected by ELISA kits.WT and Nlrp3-/- BMDMs were primed with LPS and then stimulated with Imi, Ami, Pro and Nor, the levels of IL-1βand TNF-αwere evaluated by ELISA kits. Data are expressed as the mean ± SEM,; *P < 0.05, **P < 0.01, ***P < 0.001 vs. the control; ns, not significant; unpaired Student’s t-testfollowed by the Dunnett's post hoc test. Fig. S6. Multiple TCAs induce the NLR [file 12964_2023_1128_MOESM1_ESM.docx]

**Tricyclic antidepressants induce liver inflammation by targeting NLRP3 inflammasome activation**

Wenqing Mu^a,b,c,1^, Guang Xu^a,c,d,1,^*, Zhilei Wang^a,e,1^, Qiang Li^a,d^, Siqiao Sun^a,d^, Qin Qin^a,d^, Zhiyong Li^a,d^, Wei Shi^a,d^, Wenzhang Dai^a,d^, Xiaoyan Zhan^a,d^, Jiabo Wang^a,d^, Zhaofang Bai^a,d,^**, Xiaohe Xiao^a,d,^***

^a^ Department of Hepatology, the Fifth Medical Center of PLA General Hospital, Beijing, 100039, China.

^b^ State Key Laboratory of Radiation Medicine and Protection, Institutes for Translational Medicine, Soochow University, Suzhou, Jiangsu, 215123, China.

^c^ School of Traditional Chinese Medicine, Capital Medical University, Beijing 100069, China.

^d^ Military Institute of Chinese Materia, Fifth Medical Center of Chinese PLA General Hospital, Beijing, 100039, China.

^e^ TCM Regulating Metabolic Diseases Key Laboratory of Sichuan Province, Hospital of Chengdu University of Traditional Chinese Medicine, Chengdu, 610072, China

^1^ These authors contribute equally to the work.

*****Corresponding author:** Xiaohe Xiao, Department of Hepatology, the Fifth Medical Center of PLA General Hospital, Beijing, 100039, China. [Tel: 86.010.6693.3325](Tel:+86-933325). Email: pharmacy_302@126.com.

****Corresponding author:** Zhaofang Bai, Department of Hepatology, the Fifth Medical Center of PLA General Hospital, Beijing, 100039, China. [Tel: 86.010.6693.3325](Tel:+86-933325). Email: [baizf2008@hotmail.com](mailto:baizf2008@hotmail.com).

***Corresponding author:** Guang Xu, Department of Hepatology, the Fifth Medical Center of PLA General Hospital, Beijing, 100039, China. [Tel: 86.010.6693.3325](Tel:+86-933325). Email: guang_xu@ccmu.edu.cn.


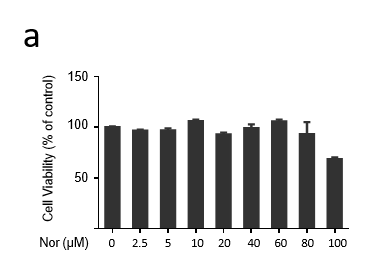


**Fig. S1.** (**a**) Cell viability of BMDMs administrated with nortriptyline was assessed using CellTiter-Glo Assay, which is according to quantitation of ATP.


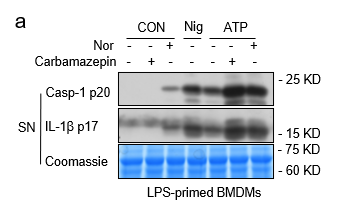


**Fig. S2. Tricyclic antidepressant (TCA) nortriptyline triggers the inflammasome activation in the absence of agonists.** (a) LPS-primed BMDMs were treated with carbamazepine and nortriptyline for 12 h or treated with them for 1 h followed by ATP stimulation, respectively. Western blotting was used to assess the expression of caspase-1 and IL-1β in SN.


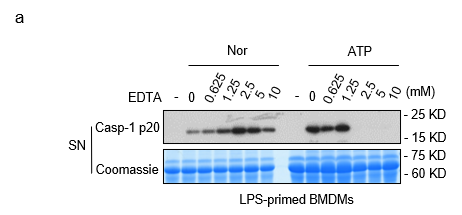


**Fig. S3.** **Nortriptyline has no effect on Ca^2+^ mobilization. (a)** LPS-primed BMDMs were pretreated with EDTA (0.625, 1.25, 2.5, 5, 10 mM) and then stimulated with nortriptyline. Western blotting was used to assess the expression of caspase-1 in SN.


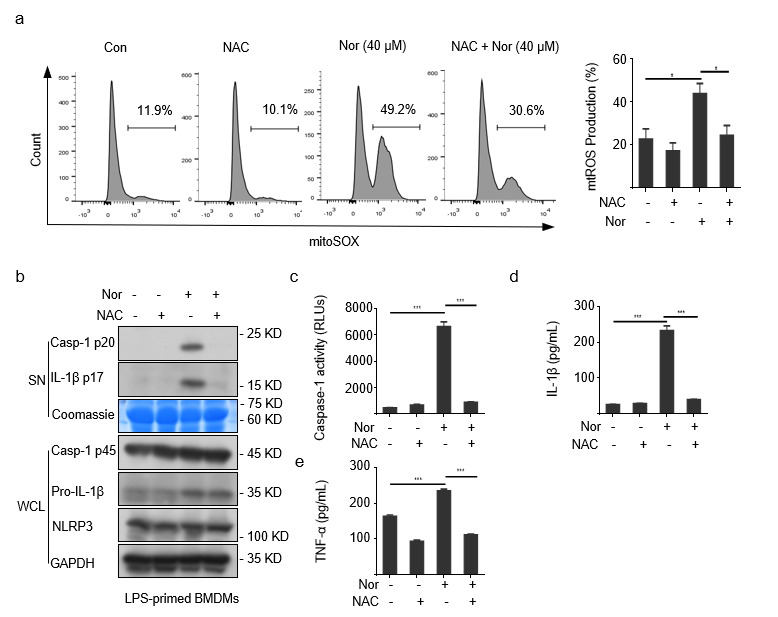


**Fig. S4. Nortriptyline activates NLRP3 inflammasome by inducing the accumulation of mtROS.** (**a–e**) LPS-primed BMDMs were pretreated with NAC (2.5 mM) for 1 h and then treated with nortriptyline for 6 h. The content of mtROS (**a**) was measured by flow cytometry. Western blotting was used to assess the expression of IL-1β and caspase-1 in cell SN as well as pro-IL-1β, pro-caspase-1, and NLRP3 (**b**) in WCL. The level of caspase-1 (**c**) activity. The levels of IL-1β (**d**) and TNF-α (**e**) in SN using ELISA. Data are presented as mean ± SEM (n= 3); **P* < 0.05, ****P* < 0.001; One-Way ANOVA followed by Dunnett's post hoc test.


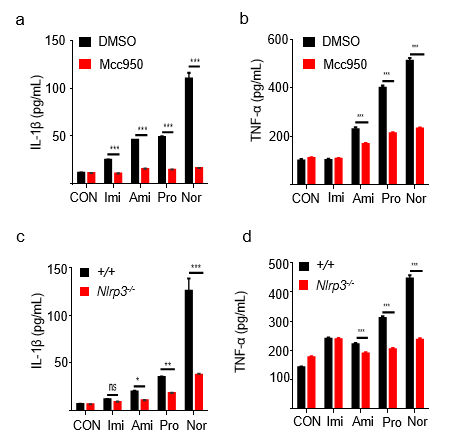


**Fig. S5. Multiple TCAs specifically trigger the aberrant activation of NLRP3 inflammasome.** (**a** and **b**) LPS-primed BMDMs were pretreated with MCC950 and then stimulated with Imi, Ami, Pro, and Nor for 12 h, the levels of IL-1β (**a**) and TNF-α (**b**) were detected by ELISA kits. (**c** and **d**) WT and *Nlrp3^-/-^* BMDMs were primed with LPS and then stimulated with Imi, Ami, Pro and Nor, the levels of IL-1β (**c**) and TNF-α (**d**) were evaluated by ELISA kits. Data are expressed as the mean ± SEM, (n = 3); **P* < 0.05, ***P* < 0.01, ****P* < 0.001 *vs.* the control; ns, not significant; unpaired Student’s *t*-test (two groups) followed by the Dunnett's post hoc test (multi groups).


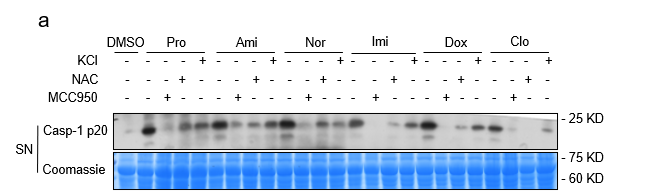


**Fig. S6. Multiple TCAs induce the NLRP3 inflammasome activation by triggering upstream signaling events.** (**a**) LPS-primed BMDMs were pretreated with MCC950, NAC, and KCl for 1 h followed by these TCAs stimulation, respectively. Western blotting was used to assess the expression of caspase-1in SN.


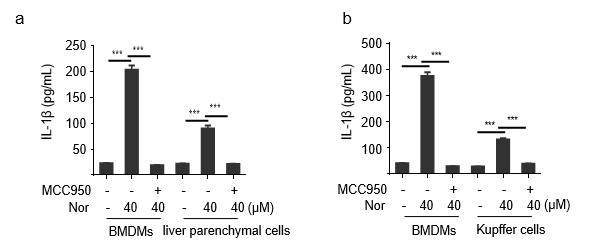


**Fig. S7. Nortriptyline directly triggered activation of the NLRP3 inflammasome in both hepatocytes and Kupffer cells.** (**a**) LPS-primed BMDMs and hepatocytes were pretreated with MCC950 and then stimulated with nortriptyline for 12 h. The levels of IL-1β in SN were detected using ELISA kits. (**b**) BMDMs and Kupffer cells were primed with LPS and then pretreated with MCC950 followed by nortriptyline stimulation for 12 h. The levels of IL-1β in SN were detected using ELISA kits. Data are presented as the mean ± SEM (n = 3); ****P* < 0.001; One-Way ANOVA followed by the Dunnett's post hoc test.


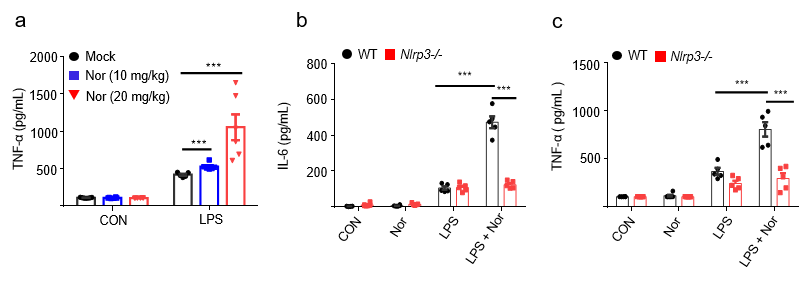


**Fig. S8. Nortriptyline induces IDILI by triggering the activation of NLRP3 inflammasome.** (**a**) Female WT C57BL/6 mice were pretreated with LPS (2 mg/kg) and then treated with different doses of nortriptyline (10 mg/kg, 20 mg/kg; n=7 control group; n=5 LPS group; n=6 other groups). The serum levels of TNF-α were detected by ELISA kits. (**b** and **c**) WT or *Nlrp3^-/-^* mice were pretreated with LPS and then treated with nortriptyline ( WT mice: n=6 control group and nortriptyline group; n=5 other WT groups; *Nlrp3^-/-^* mice: n=6 control group; n=5 other *Nlrp3^-/-^* groups). ELISA kits were used to determine the levels of IL-6 (**b**) and TNF-α (**c**) in mouse sera. Data are shown as the mean ± SEM; ****P* < 0.001; ns, not significant. Statistics differences were analyzed using an unpaired Student’s t-test.
