# Supplementary material for: Tricyclic antidepressants induce liver inflammation by targeting NLRP3 inflammasome activation
Source: Cell Commun Signal. 2023 May 25;21:123. doi: 10.1186/s12964-023-01128-x (PMC10214596; doi:10.1186/s12964-023-01128-x)
Supplement: Supplementary file 3 — Additional file 2: Raw data. [file 12964_2023_1128_MOESM2_ESM.docx]

**Tricyclic antidepressants induce liver inflammation by targeting NLRP3 inflammasome activation**

Wenqing Mu^a,b,c,1^, Guang Xu^a,c,d,1,^*, Zhilei Wang^a,e,1^, Qiang Li^a,d^, Siqiao Sun^a,d^, Qin Qin^a,d^, Zhiyong Li^a,d^, Wei Shi^a,d^, Wenzhang Dai^a,d^, Xiaoyan Zhan^a,d^, Jiabo Wang^a,d^, Zhaofang Bai^a,d,^**, Xiaohe Xiao^a,d,^***

^a^ Department of Hepatology, the Fifth Medical Center of PLA General Hospital, Beijing, 100039, China.

^b^ State Key Laboratory of Radiation Medicine and Protection, Institutes for Translational Medicine, Soochow University, Suzhou, Jiangsu, 215123, China.

^c^ School of Traditional Chinese Medicine, Capital Medical University, Beijing 100069, China.

^d^ Military Institute of Chinese Materia, Fifth Medical Center of Chinese PLA General Hospital, Beijing, 100039, China.

^e^ TCM Regulating Metabolic Diseases Key Laboratory of Sichuan Province, Hospital of Chengdu University of Traditional Chinese Medicine, Chengdu, 610072, China

^1^ These authors contribute equally to the work.

*****Corresponding author:** Xiaohe Xiao, Department of Hepatology, the Fifth Medical Center of PLA General Hospital, Beijing, 100039, China. [Tel: 86.010.6693.3325](Tel:+86-933325). Email: pharmacy_302@126.com.

****Corresponding author:** Zhaofang Bai, Department of Hepatology, the Fifth Medical Center of PLA General Hospital, Beijing, 100039, China. [Tel: 86.010.6693.3325](Tel:+86-933325). Email: [baizf2008@hotmail.com](mailto:baizf2008@hotmail.com).

***Corresponding author:** Guang Xu, Department of Hepatology, the Fifth Medical Center of PLA General Hospital, Beijing, 100039, China. [Tel: 86.010.6693.3325](Tel:+86-933325). Email: guang_xu@ccmu.edu.cn.


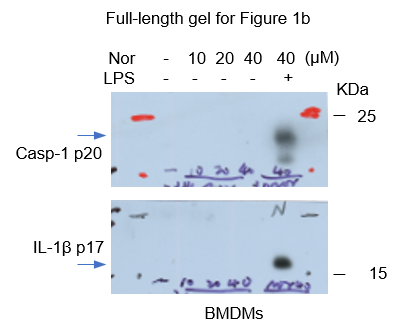


**Figure. 1b. The effect of nortriptyline on inflammasome activation in the presence or absence of LPS.** (**b**) In the presence or absence of LPS, BMDMs were stimulated with nortriptyline for 12 h. Western blotting was used to assess the expression of caspase-1 and IL-1β in cell supernatants (SN).


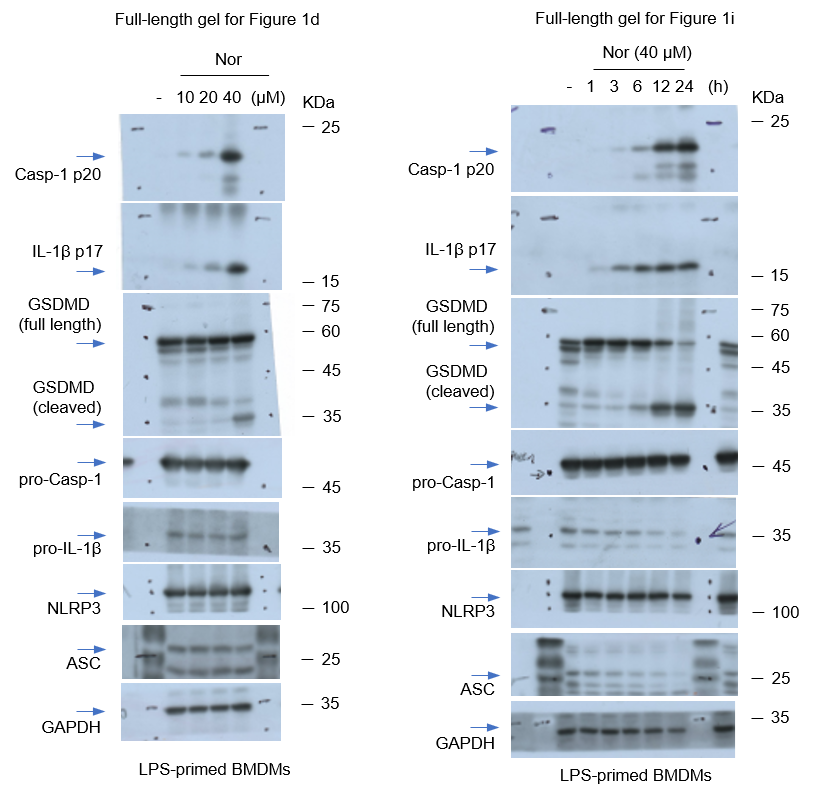


**Figure. 1d and i. Nortriptyline triggers the inflammasome activation in a dose- and time-dependent manner.** (**d**) LPS-primed BMDMs were stimulated with a range of nortriptyline concentrations. Western blot assessed the expression of mature caspase-1 and IL-1β in SN and GSDMD cleavage, pro-caspase-1, pro-IL-1β, NLRP3, and ASC in whole-cell lysates (WCL). (**i**) Cells were incubated with LPS and then treated with nortriptyline for 1, 3, 6, 12 h, respectively. Western blot evaluated the expression of mature IL-1β and cleaved caspase-1 in SN and GSDMD cleavage, pro-caspase-1, pro-IL-1β, NLRP3, and ASC in WCL.

**
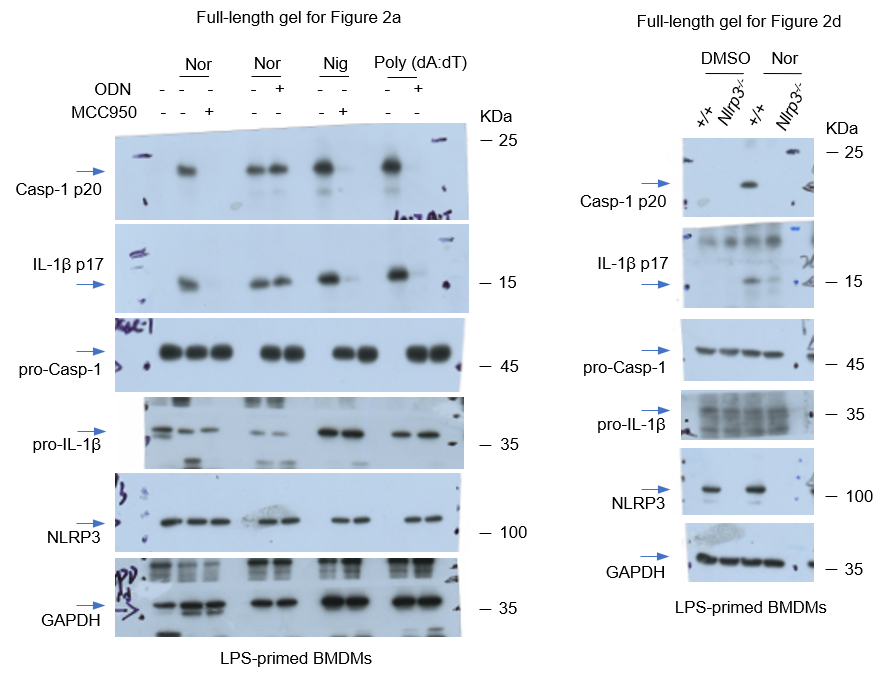
**

**Figure. 2a and d. Nortriptyline specifically activates NLRP3 inflammasome, rather than AIM2.** (**a**) Cells were incubated with LPS and then pretreated with MCC950 or ODN followed by nortriptyline treatment. Western blot assessed the expression of caspase-1 and IL-1β in SN and pro-caspase-1, pro-IL-1β, and NLRP3 in WCL. (**d**) *Nlrp3^-/-^* BMDMs were incubated with LPS and nortriptyline. The expression of IL-1β and caspase-1 in SN and pro-caspase-1, pro-IL-1β, and NLRP3 in WCL were evaluated by western blot.


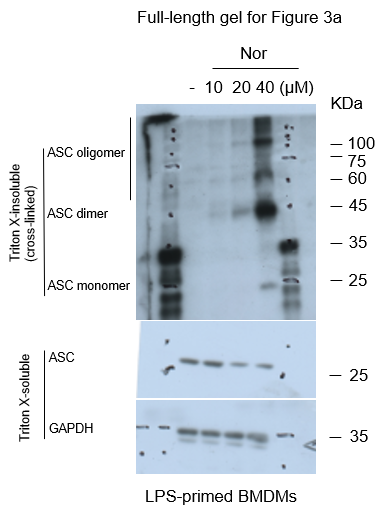


**Figure. 3a. Nortriptyline induces NLRP3-dependent ASC oligomerization.** (**a**) The ASC oligomerization from BMDMs stimulated with nortriptyline was evaluated by western blot.


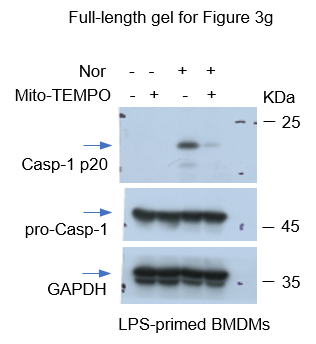


**Figure. 3g. Nortriptyline induces the NLRP3 inflammasome activation by triggering mtROS accumulation.** (**g**) BMDMs were incubated with LPS and Mito-TEMPO followed by nortriptyline treatment, the expression of caspase-1 in SN and pro-caspase-1 and pro-IL-1β in WCL was assessed via western blotting.


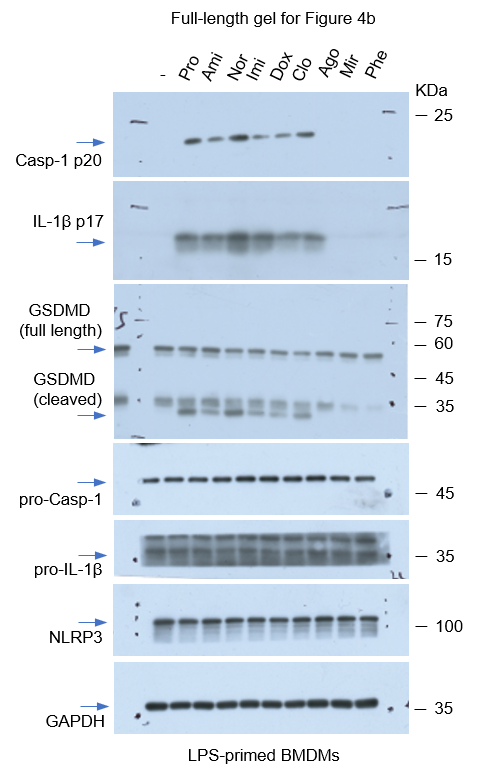


**Figure. 4b. Multiple tricyclic antidepressants trigger the inflammasome activation.** (**b**) LPS-primed BMDMs were stimulated with protriptyline, amitriptyline, nortriptyline, imipramine, doxepin, clomipramine, agomelatine, mirtazapine, and phenothiazine. Western blot assessed the expression of caspase-1 and IL-1β in SN and GSDMD cleavage, pro-caspase-1, pro-IL-1β, and NLRP3 in WCL.


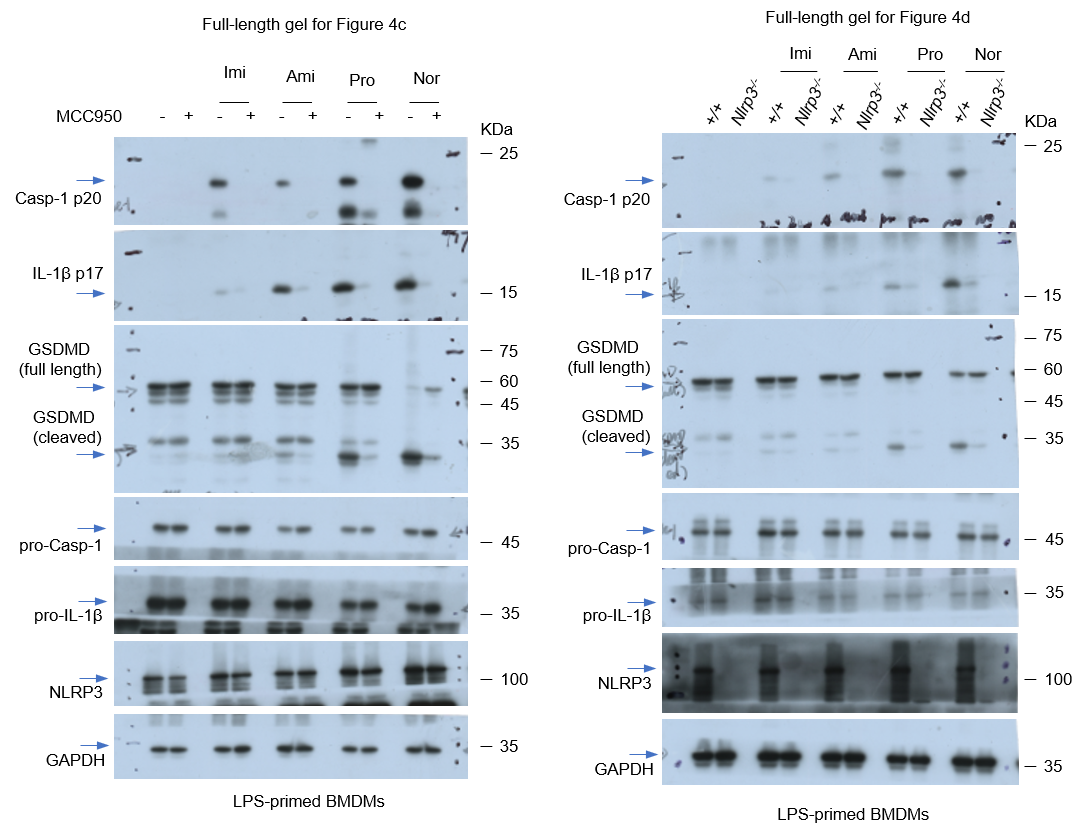


**Figure. 4c and d.** **Multiple tricyclic antidepressants specifically induce the NLRP3 inflammasome activation.** (**c**) BMDMs were incubated with LPS and MCC950 and then treated with imipramine, amitriptyline, protriptyline and nortriptyline, respectively. The expressions of IL-1β and caspase-1 in SN as well as GSDMD cleavage, pro-caspase-1, pro-IL-1β, and NLRP3 in WCL were evaluated using western blotting. (**d**) *Nlrp3^-/-^* BMDMs were incubated with LPS followed by imipramine, amitriptyline, protriptyline and nortriptyline stimulation. The expression of IL-1β and caspase-1 in SN and GSDMD cleavage, pro-caspase-1, pro-IL-1β, and NLRP3 in WCL were measured by western blotting.


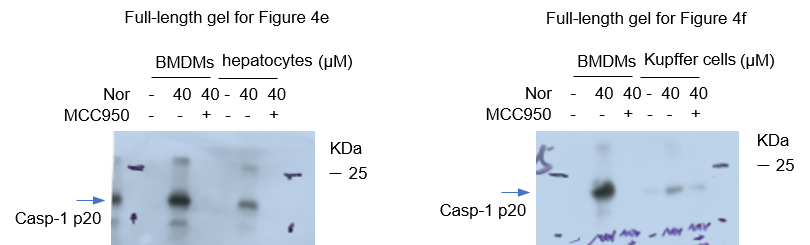


**Figure. 4e and f. Nortriptyline induces the NLRP3 inflammasome activation in liver cells.** (**e**) BMDMs or hepatocytes were pretreated with LPS and MCC950 and then treated with nortriptyline, western blot was used to measure the expression of caspase-1 in SN. (**f**) BMDMs or Kupffer cells were incubated with LPS and MCC950 followed by nortriptyline treatment. The expression of caspase-1 in SN were assessed by western blot.


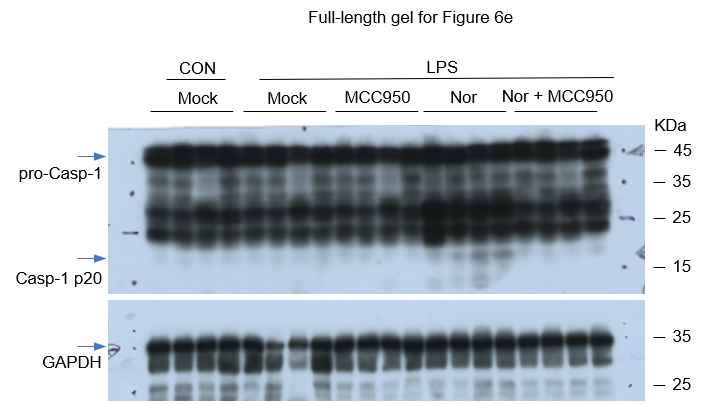


**Figure. 6e. MCC950 pretreatment rescues nortriptyline-driven liver toxicity.** (**e**) WT mice were pretreated with MCC950 followed by LPS treatment and then stimulated with nortriptyline. Western blot analysis of caspase-1 activation of the liver tissue.


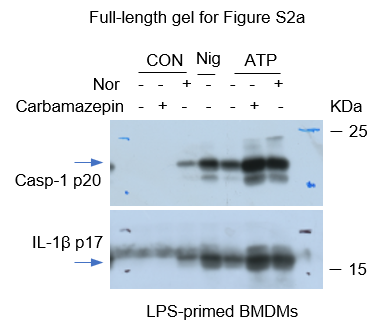


**Figure. S2a. Tricyclic antidepressant nortriptyline triggers the inflammasome activation in the absence of agonists.** (**a**) LPS-primed BMDMs were treated with carbamazepine and nortriptyline for 12 h or treated with them for 1 h followed by ATP stimulation, respectively. Western blotting was used to assess the expression of caspase-1 and IL-1β in SN.


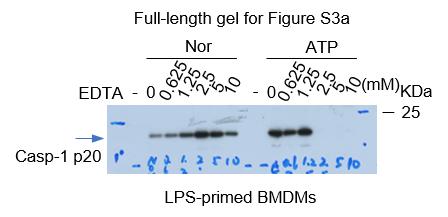


**Figure. S3a. Nortriptyline has no effect on Ca^2+^ mobilization. (a)** LPS-primed BMDMs were pretreated with EDTA (0.625, 1.25, 2.5, 5, 10 mM) and then stimulated with nortriptyline. Western blotting was used to assess the expression of caspase-1 in SN.


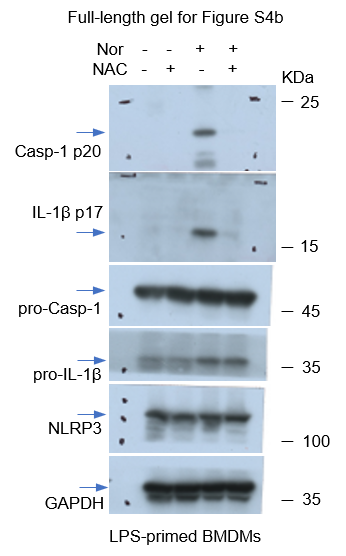


**Figure. S4b. Nortriptyline activates NLRP3 inflammasome by inducing the accumulation of mtROS.** (**b**) LPS-primed BMDMs were pretreated with NAC (2.5 mM) for 1 h and then treated with nortriptyline for 6 h. Western blotting was used to assess the expression of IL-1β and caspase-1 in cell SN as well as pro-IL-1β, pro-caspase-1 and NLRP3 in WCL.


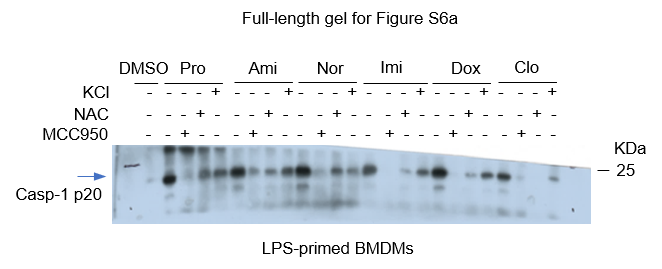


**Figure. S6a. Multiple TCAs induce the NLRP3 inflammasome activation by triggering upstream signaling events.** (**a**) LPS-primed BMDMs were pretreated with MCC950, NAC, and KCl for 1 h followed by these TCAs stimulation, respectively. Western blotting was used to assess the expression of caspase-1in SN.
